# Comparative Physiological Analysis of Methyl Jasmonate in the Delay of Postharvest Physiological Deterioration and Cell Oxidative Damage in Cassava

**DOI:** 10.3390/biom9090451

**Published:** 2019-09-05

**Authors:** Guoyin Liu, Bing Li, Xiuqiong Li, Yunxie Wei, Debing Liu, Haitao Shi

**Affiliations:** 1 Hainan Key Laboratory for Sustainable Utilization of Tropical Bioresources, College of Tropical Crops, Hainan University, Haikou 570228, China (G.L.) (B.L.); 2 College of Forestry, Hainan University, Haikou 570228, China; 3 College of Applied Science and Technology, Hainan University, Danzhou 571737, China

**Keywords:** cassava, methyl jasmonate, postharvest physiological deterioration, cell oxidative damage, reactive oxygen species

## Abstract

The short postharvest life of cassava is mainly due to its rapid postharvest physiological deterioration (PPD) and cell oxidative damage, however, how to effectively control this remains elusive. In this study, South China 5 cassava slices were sprayed with water and methyl jasmonate (MeJA) to study the effects of MeJA on reactive oxygen species, antioxidant enzymes, quality, endogenous hormone levels, and melatonin biosynthesis genes. We found that exogenous MeJA could delay the deterioration rate for at least 36 h and alleviate cell oxidative damage through activation of superoxide dismutase, catalase, and peroxidase. Moreover, MeJA increased the concentrations of melatonin and gibberellin during PPD, which had a significant effect on regulating PPD. Notably, exogenous MeJA had a significant effect on maintaining cassava quality, as evidenced by increased ascorbic acid content and carotenoid content. Taken together, MeJA treatment is an effective and promising way to maintain a long postharvest life, alleviate cell oxidative damage, and regulate storage quality in cassava.

## 1. Introduction

Cassava (*Manihot esculenta*) is one of the most important tropical crops [1,2]. Due to the short postharvest shelf-life of cassava, its potential market benefit to cassava farmers is severely restricted. Postharvest physiological deterioration (PPD) is harmful during harvesting and safekeeping, and the burst of reactive oxygen species (ROS) results in serious cell oxidative damage [2,3,4,5]. ROS is regulated by an antioxidant system including enzymatic and non-enzymatic scavenging mechanisms [6]. The enzymatic scavenging mechanism mainly consists of catalase (CAT), superoxide dismutase (SOD), ascorbate peroxidase (APX), and peroxidase (POD), while the non-enzymatic scavenging mechanism includes reduced forms of ascorbate, carotenoids, and flavonoids [7].

So far, various treatments have paid more attention to inhibiting cassava PPD. For example, hot water treatment for 10 min and modified atmosphere packaging have significant effects on delaying PPD during cassava postharvest storage [8]. In addition, keeping the cassava at 10 °C and 80% relative humidity could also delay PPD for 14 d [9]. Meanwhile, exogenous treatments with some small molecules, such as melatonin [10,11,12] and CaCl_2_ [12], can delay the postharvest shelf-life of cassava root. Although traditional or genetic breeding methods can inhibit PPD in cassava [13,14], these methods still have many problems such as long-term breeding, biosafety, and others [1]. Generally, all the above methods have both advantages and shortcomings. Therefore, other suitable techniques for delaying or inhibiting PPD in cassava need to be further investigated.

As an important plant growth regulator, methyl jasmonate (MeJA) has significant effects on quality [15,16], fruit ripening, senescence, and underlying parameters [17,18]. Previous studies have demonstrated that MeJA treatment can induce the expression levels of SOD, POD, and CAT [17,18] to decrease the levels of hydrogen peroxide (H_2_O_2_) and superoxide anion (O_2_^−^) [19], thereby improving stress resistance [17]. MeJA treatment is also conducive to the maintenance of eggplant quality during storage, inhibiting the weight loss and calyx browning [18]. Postharvest treatment with MeJA maintains higher concentrations of sugars and organic acids in fresh kiwifruit [20] and mangoes [21]. Moreover, the application of MeJA to fruits and vegetables can increase the accumulation of flavonoids [22,23,24] and quality [25,26,27]. Additionally, soluble solids can also be significantly induced by MeJA treatment [28].

Although MeJA has significant effects on fruit ripening and storage quality, the direct correlation between MeJA and PPD in cassava remains unclear. In this study, comparative physiological analysis was performed to reveal the effect of MeJA in PPD, cell oxidative damage, and cassava quality. Notably, the relationship between MeJA and melatonin during cassava PPD was also revealed.

## 2. Materials and Methods

### 2.1. Plant Materials and Treatments

*Manihot esculenta* Crantz. cv. Mainland South China 5 (SC5) cassava roots were harvested from nine-month-old cassava in Baisha County, Hainan Province, China. The cassava roots were washed with double distilled water, the proximal and distal parts of the cassava roots were eliminated, and the remaining roots were cut to 5–10 mm thick cassava slices. The cassava slices were randomly divided into nine treatments and each treatment included 45 cassava slices. Cassava slices were sprayed with either mock (the control having the same pH as the other treatments), 20 μM MeJA, 0.5 mM MeJA, 5 mM MeJA, 10 mM MeJA, 20 μM gibberellin (GA), 2.5 mM GA, 5 mM GA, or 10 mM GA as different treatments, and kept at 25 °C with 60%–75% relative humidity. The cassava root slices were gathered at 0, 12, 24, and 48 h, then frozen in liquid nitrogen and stored at −80 °C for subsequent determination. For the reagents, MeJA (39924-52-2, purity ≥ 95%, Solarbio, Beijing, China) and GA (77-06-5, purity ≥ 90%, Biotopped, Beijing, China) were used.

### 2.2. Visual PPD Evaluation

Visual inspection of each slice was conducted at 0, 12, 24, 36, 48 and 72 h. The vascular discoloration was quantified as determined percentages using ImageJ analysis software (http://rsb.info.nih.gov/ij/). The percentages of gray values indicate the deterioration rate at each time point by the software. The gray value at 0 h was set to 1.

### 2.3. Assays of ROS Accumulation and Antioxidant Enzyme Activities

The endogenous ROS accumulation and antioxidant enzyme activities were determined with a microplate reader. Briefly, 0.5 g of root slices were taken and mixed with 5 mL with a 50 μM phosphate buffer (pH 7.8). After centrifuged at 12,000× *g* for 10 min at 4 °C, the supernatant was collected for determination of H_2_O_2_ and O_2_^−^ content as well as enzyme activities. The content of O_2_^−^ was determined according to the hydroxylamine reaction method. The content of H_2_O_2_ and activities of CAT, POD, and SOD were detected using the H_2_O_2_ Assay Kit (A064, Jiancheng, Nanjing, China), CAT Assay Kit (A007-1, Jiancheng, Nanjing, China), POD Assay Kit (A084-3, Jiancheng, Nanjing, China), and SOD Assay Kit (A001-4, Jiancheng, Nanjing, China), respectively, according to the manufacturer’s guidelines.

### 2.4. RNA Isolation and Quantitative Real-Time PCR (qRT-PCR)

Total RNA isolation and qRT-PCR was performed according to the manufacturer’s guidelines as described by Wei et al. [29]. The protocol of qRT-PCR included 95 °C for 10 min, followed by 45 cycles of 95 °C for 30 s, 55 °C for 30 s, and 72 °C for 30 s. Then the relative transcription levels were evaluated using the comparative 2^−ΔΔCT^ method with *MeEF1* as the reference gene. The primers have been previously described [30].

### 2.5. Determination of Endogenous Melatonin and GA

The endogenous melatonin and GA were quantified using the Melatonin Enzyme-Linked Immunosorbent Assay (ELISA) Kit (HLE97243, Haling Biotechnology, Shanghai, China) and GA ELISA Kit (HLE97151, Haling Biotechnology, Shanghai, China) respectively, according to the manufacturer’s protocols.

### 2.6. Quantification of Starch, Soluble Sugar, Ascorbic Acid, and Carotenoid

The level of ascorbic acid was determined using the Ascorbic Acid Assay Kit (A009, Jiancheng, Nanjing, China). The concentrations of soluble sugar and carotenoid were measured as described by Gao [31]. Briefly, the homogenate was extracted twice in 5 mL of 80% ethanol at 80 °C for 30 min. After centrifugation at 12,000× *g* for 10 min at room temperature, the supernatant was collected for the determination of soluble sugar by an anthrone colorimeter. Carotenoid was extracted from 0.5 g of cassava root slices using 5 mL of 96% ethanol, and the absorbance was determined at 665, 649, and 470 nm, using a microplate reader and a 96-well plate. Meanwhile, starch concentration was determined as described by Cao et al. [32]. For starch determination, the homogenate was extracted twice in 5 mL of 80% ethanol at 80 °C for 30 min, residuals were gelatinized at 100 °C for 15 min, then 2 mL of 9.2 mol/L perchloric acid was added for further extraction for 15 min. After being centrifuged at 12,000× *g* for 10 min at room temperature, the supernatant was collected for the determination of starch.

### 2.7. Statistical Analysis

All experiments were performed with at least three independent biological replicates. All data were shown as means ± SD, and were analyzed using ANOVA and SAS 9.1.3 statistics software (9.1.3, SAS Instituteinc, North Carolina, NC, USA) for Duncan’s multiple range test. Asterisk symbols (*) indicated a significant difference at *p* < 0.05 at the same time.

## 3. Results

### 3.1. The Effect of MeJA Treatment on PPD

**Figure 1:**
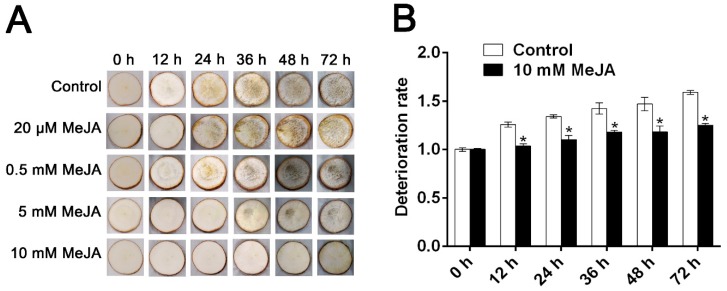
The effects of methyl jasmonate (MeJA) on postharvest physiological deterioration (PPD) in cassava storage root slices of South China 5 (SC5) variety. Visual detection (**A**) and deterioration rate (**B**) of storage root slices during PPD. Deterioration rate was determined by the percentages of gray values at different time points. Data are means ± SD calculated from at least three biological replicate samples. Asterisk symbols (*) indicate significant differences according to Duncan’s multiple range test at *p* < 0.05 at the same time points.

As shown in Figure 1A, the effect of MeJA on postharvest physiological deterioration (PPD) symptoms of cassava storage roots was revealed. In addition, the deterioration rate is shown in Figure 1B. Obviously, higher concentration of MeJA (5 mM and 10 mM) significantly delayed the development of PPD and decreased deterioration rate (Figure 1). Thus, 10 mM MeJA was selected for further study.

After 12 h of water treatment, a brown color symptom was observed in the cassava root slices. However, the symptom in question did not manifest until 72 h with 10 mM MeJA treatment (Figure 1A). On the contrary, the untreated cassava root slices displayed a high deterioration rate (Figure 1B). The results obviously showed that the deterioration rate of root slices was gradually increased during storage time, and 10 mM MeJA delayed early development of PPD in root slices (Figure 1). These results indicated that exogenous application of MeJA could delay the occurrence of PPD in cassava root slices.

### 3.2. MeJA Alleviates Cell Oxidative Damage through Modulation of ROS and Underlying Antioxidant Enzymes

**Figure 2:**
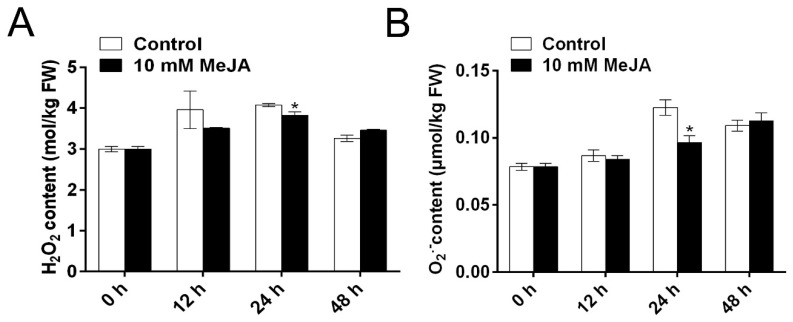
MeJA alleviates cell oxidative damage through modulation of reactive oxygen species (ROS) in cassava tuberous roots during PPD. The concentrations of H_2_O_2_ content (**A**) and O_2_^−^ content (**B**) of cassava tuberous roots. Data are means ± SD calculated from three biological replicate samples. Asterisk symbols (*) indicate significant differences according to Duncan’s multiple range test at *p* < 0.05 at the same time points.

**Figure 3:**
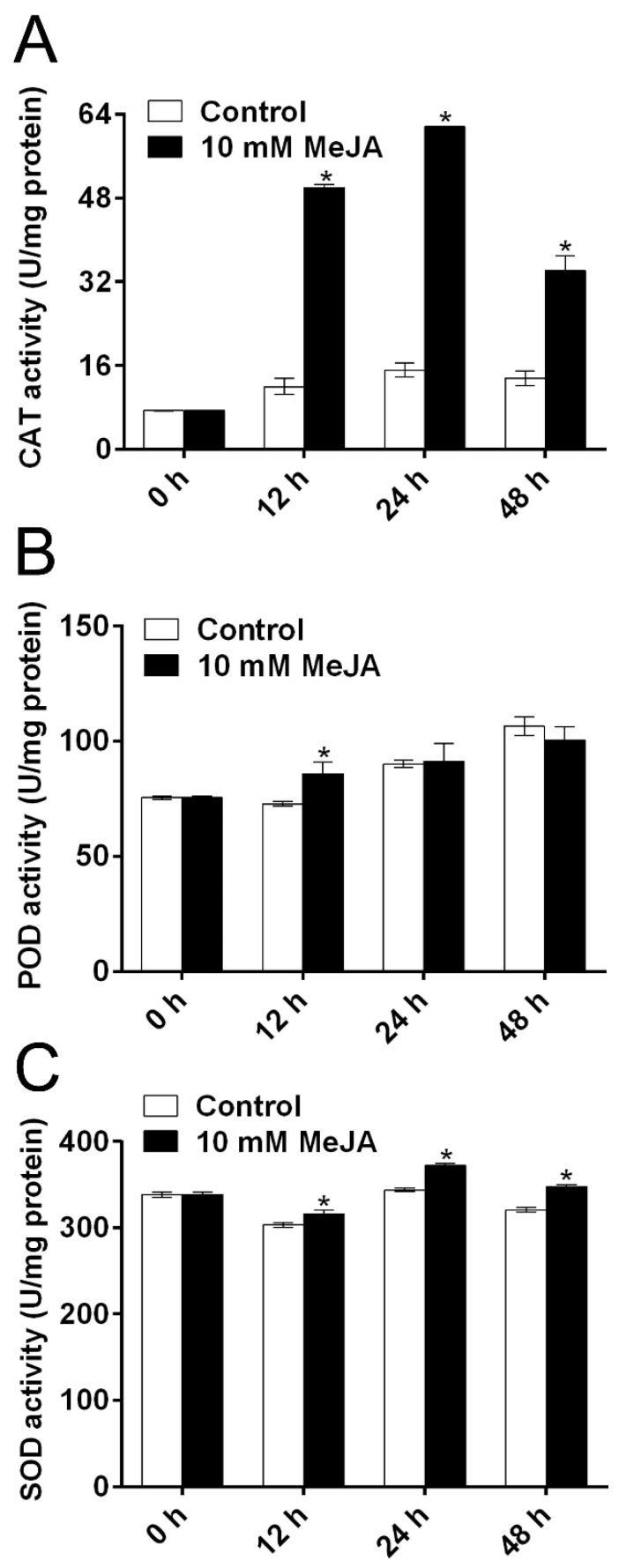
The modulation of MeJA treatment on antioxidant enzyme activities in cassava tuberous roots during PPD. Related activities of catalase (CAT) (**A**), peroxidase (POD) (**B**), and superoxide dismutase (SOD) (C) in cassava tuberous roots. Data are means ± SD calculated from at least four biological replicate samples. Asterisk symbols (*) indicate significant differences according to Duncan’s multiple range test at *p* < 0.05 at the same time.

To explore whether MeJA-induced delay of PPD was related to ROS scavenging in cassava root slices during the storage period, the concentrations of H_2_O_2_ and O_2_^−^ were determined at different time points (Figure 2). Moreover, the concentrations of H_2_O_2_ and O_2_^−^ in MeJA-treated cassava were significantly lower than that of untreated cassava at 24 h, while there was no significant difference at other time points. Antioxidant enzymes play vital roles in scavenging ROS and alleviating oxidative damage under stress environment [33,34]. The activities of CAT and SOD in MeJA-treated root slices were significantly higher than those in the untreated root slices at 12, 24, and 48 h (Figure 3A,C). Although the activity of POD in MeJA-treated cassava root slices was significantly higher than that in untreated cassava root slices at 12 h, there were no significant differences at other time points (Figure 3B). These results indicated that 10 mM MeJA could alleviate cell oxidative damage through activating the activities of antioxidant enzymes, which might contribute to the burst of H_2_O_2_ and O_2_^−^ during storage time.

### 3.3. MeJA Positively Modulates the Quality of Cassava Root Slices

**Figure 4:**
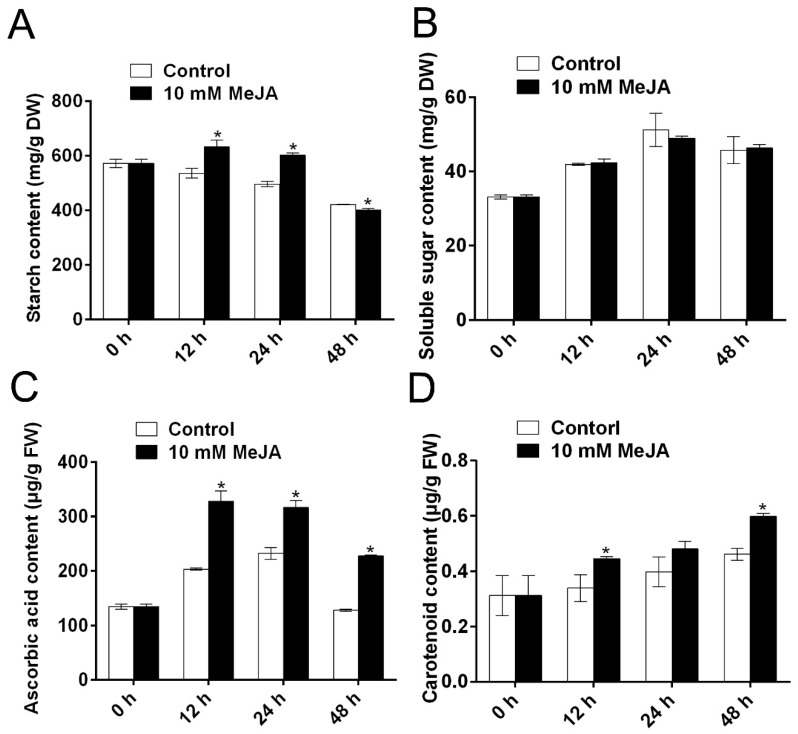
The modulation of MeJA treatment on the quality of cassava tuberous roots during PPD. The concentrations of starch content (**A**), soluble sugar content (**B**), ascorbic acid content (**C**), and carotenoid content (**D**) in cassava tuberous roots during PPD progression. Data are means ± SD calculated from three biological replicate samples. Asterisk symbols (*) indicate significant differences according to Duncan’s multiple range test at *p* < 0.05 at the same time.

During the process of PPD, starch concentration was significantly higher in MeJA-treated cassava root slices than that in the control root slices at 12 and 24 h, while at 48 h, a decrease was evident after MeJA treatment (Figure 4A). Soluble sugar concentration showed no significant difference between MeJA-treated cassava root slices and the control root slices (Figure 4B). Furthermore, MeJA treatment significantly increased ascorbic acid levels in comparison to the control during cassava slice storage (Figure 4C), and MeJA treatment significantly increased carotenoid concentration compared with the control, at 12 and 48 h (Figure 4D). These quality-related parameters showed that MeJA could reduce quality loss in cassava root slices during storage.

### 3.4. MeJA Treatment Affects the Endogenous GA Content

**Figure 5:**
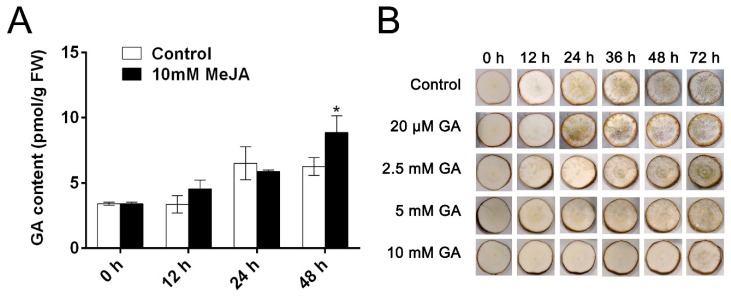
The effects of MeJA on the endogenous gibberellin (GA) levels in stored cassava slices. (**A**) The endogenous levels of GA in cassava storage root slices without and with MeJA pre-treatment. (**B**) Visual detection of storage root slices affected by GA pre-treatment. Data are means ± SD calculated from three biological replicate samples. Asterisk symbols (*) indicate significant differences according to Duncan’s multiple range test at *p* < 0.05 at the same time.

Interestingly, MeJA treatment increased endogenous GA concentration as compared to the control at 12 and 48 h during cassava storage (Figure 5A). Simultaneously, a high concentration of GA also significantly delayed the development of PPD (Figure 5B). These results demonstrated that MeJA-induced GA also had a significant effect on delaying PPD.

### 3.5. The Effect of MeJA on the Expression of Melatonin Biosynthesis Genes and Melatonin Level

**Figure 6:**
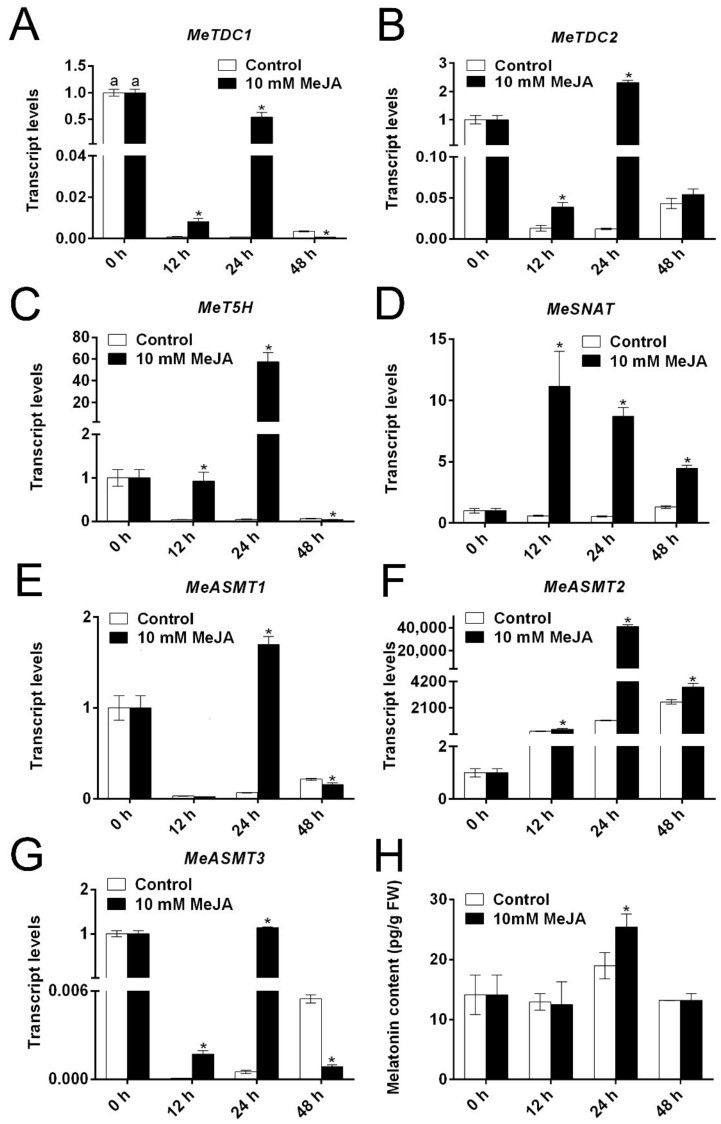
The effects of the MeJA on the expression levels of cassava melatonin biosynthesis genes and underlying endogenous melatonin. Expression levels of *MeTDC1* (**A**), *MeTDC2* (**B**), *MeT5H* (**C**), *MeSNAT* (**D**), *MeASMT1* (**E**), *MeASMT2* (**F**), and *MeASMT3* (**G**) during PPD progression. The endogenous melatonin level in cassava storage root slices (**H**). Data are means ± SD calculated from three biological replicate samples. Asterisk symbols (*) indicate significant differences according to Duncan’s multiple range test at *p* < 0.05 at the same time.

Besides GA, the endogenous melatonin level and the corresponding melatonin biosynthesis relative genes were also determined. Notably, all the genes except *MeASMT1* were significantly upregulated after MeJA treatment in comparison to the control root slices at 12 h (Figure 6). Compared with the control samples, all these genes were significantly upregulated at 24 h after MeJA treatment (Figure 6). Meanwhile, the endogenous melatonin level also significantly increased after MeJA treatment at 24 h (Figure 6H). Therefore, exogenous MeJA might act as a molecular regulator to activate melatonin biosynthesis, thereby delaying cassava PPD and alleviating cell oxidative damage.

## 4. Discussion

In this study, the activities of SOD, CAT, and POD in MeJA-treated cassava roots were significantly higher than those in the control roots (Figure 3). Consistently, the concentrations of O_2_^−^ and H_2_O_2_ showed the opposite trend (Figure 2), thereby delaying PPD symptoms and alleviating cell oxidative damage. Previous studies have shown that the application of MeJA could increase the activities of SOD [17], POD [17,18], and CAT [17,18] in different fruits and plants, and thereby stress resistance [17]. SOD can eliminate O_2_^−^ and transform it into H_2_O_2_, which is further reduced to H_2_O by CAT [35]. CAT, as a detoxifying system member, protects cells against the ROS accumulated within cells [19]. Overall, these results indicated that MeJA might delay PPD and decrease cell oxidative damage in cassava root slices through enhancing antioxidant enzyme activities and decreasing ROS accumulation.

After MeJA pre-treatment, the concentrations of ascorbic acid, carotenoid, and soluble sugar were increased, while the degradation of starch was reduced (Figure 4). Hu et al. [12] indicated that the application of CaCl_2_ could reduce the degradation of ascorbic acid and delay PPD, in accordance with this study. In addition, Chavez et al. [36] found that there is positive correlation between endogenous β-carotene level and cassava PPD tolerance, so the modulation of MeJA on carotenoids may contribute to its effect on cassava PPD. MeJA treatment is conducive to the maintenance of eggplant fruit quality during storage, through inhibiting the increase of calyx browning [18]. MeJA treatment can maintain higher concentrations of sugars and organic acids in fresh kiwifruit [20] and mangoes [21]. The degradation of starch can result in the increase of sugar [37,38]. Soluble sugar is the basis for the formation of fruit quality. Moreover, ascorbic acid also plays an essential role in plant antioxidant stress defense and nutrition [39]. Therefore, the effects of exogenous MeJA treatment on the above parameters might be used for delaying senescence as well as maintaining storage quality.

**Figure 7:**
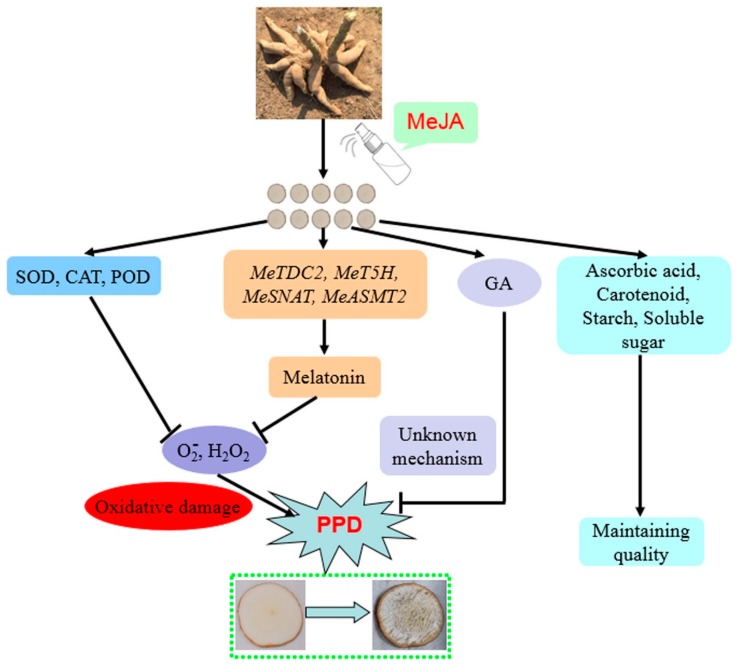
A possible model of MeJA-mediated cassava PPD.

Plant growth regulators play important roles in many physiological processes and stress responses. This study showed that the endogenous GA level was higher in MeJA-treated cassava root slices than that in the control slices (Figure 5A). Furthermore, GA treatment has significant effects on delaying ripening and postponing senescence [40,41,42]. Besides GA, exogenous melatonin treatment delayed postharvest senescence in litchi [43], banana [44], peach [45], and strawberry [46]. In cassava, melatonin biosynthesis genes are transcriptionally upregulated by melatonin treatment [10], and the application of CaCl_2_ can also increase melatonin content through activating the expression of melatonin biosynthesis genes [12]. Herein, MeJA commonly activated the transcripts of *MeTDC1/2*, *MeT5H*, *MeSNAT*, and *MeASMT1/2/3*, so as to trigger endogenous melatonin levels in cassava root slices during cassava storage. Based on the relationship between plant growth regulators and fruit ripening as well as quality, we concluded that the modulation of MeJA on ROS might contribute to MeJA-mediated PPD and cassava quality. Figure 7 shows a possible model describing the potential relationships among MeJA, melatonin, ROS, GA, quality, and cassava PPD. PPD is connected with a ROS burst and the activation of underlying antioxidant enzymes. In this study, MeJA could alleviate cell oxidative damage through modulation of ROS and underlying antioxidant enzymes, and increase the concentration of melatonin and GA, resulting in a delayed deterioration rate. Therefore, the results indicated that MeJA could delay the cassava deterioration rate through the modulation of multiple physiological parameters (Figure 7).

## 5. Conclusions

MeJA delays the deterioration rate and alleviates cell oxidative damage through modulation of ROS accumulation and the underlying activities of SOD, CAT, and POD. In addition, exogenous MeJA has a significant effect on maintaining cassava quality, including ascorbic acid and carotenoids. Therefore, MeJA treatment is an effective and promising way to maintain a long postharvest life, decrease cell damage, and regulate storage quality in cassava.
